# Prevalence of endogenous normal thyroid function 3 years after hemithyroidectomy for differentiated thyroid cancer

**DOI:** 10.1530/ETJ-24-0282

**Published:** 2025-09-11

**Authors:** Tina Toft Kristensen, Christina Caroline Plaschke, Anne Fog Lomholt, Christoffer Holst Hahn, Irene Wessel, Mads Aage Toft Kristensen, Jens Bentzen, Christina Ellervik

**Affiliations:** ^1^ Department of Otorhinolaryngology Head and Neck Surgery and Audiology, Copenhagen University Hospital – Rigshospitalet, Copenhagen, Denmark; ^2^ Department of Clinical Medicine, University of Copenhagen, Copenhagen, Denmark; ^3^ Section of General Practice, Department of Public Health, University of Copenhagen, Copenhagen, Denmark; ^4^ Department of Oncology, Herlev Hospital, Herlev, Denmark; ^5^ University of Copenhagen, Copenhagen, Denmark; ^6^ Department of Laboratory Medicine, Boston Children’s Hospital, Harvard Medical School, Boston, USA; ^7^ Department of Clinical Biochemistry, Zealand University Hospital, Køge, Denmark

**Keywords:** hemithyroidectomy, differentiated thyroid cancer, thyroid function, postoperative LT4 treatment

## Abstract

**Objective:**

To investigate the prevalence of endogenous normal thyroid function 3 years after hemithyroidectomy for low-risk differentiated thyroid cancer if a postoperative thyroid-stimulating hormone increase up to 4 mIU/L is accepted.

**Method:**

A retrospective review of a total of 162 Eastern Danish patients was conducted. Patients were initially followed up without levothyroxine treatment after hemithyroidectomy for differentiated thyroid cancer if thyroid-stimulating hormone was below 4 mIU/L, in accordance with the Danish treatment guideline. Patients’ hospital charts were reviewed, and data on the initiation of levothyroxine treatment, pre- and postoperative thyroid-stimulating hormone, recurrence, and remnant lobe nodularity were collected.

**Results:**

A total of 143/162 (88%) did not take levothyroxine before hemithyroidectomy, with a median (interquartile range) age of 53 (43–65) years; 80% were women. During follow-up, the prevalence of endogenous normal thyroid function gradually decreased to 80, 69, and 66% after 1, 2, and 3 years. Concomitantly, hypothyroidism developed with thyroid-stimulating hormone >4.0 mIU/L in 20, 31, and 34% of patients, who were replaced with levothyroxine. In patients not on levothyroxine, TSH was significantly increased within the normal range 1, 2, and 3 years after hemithyroidectomy for DTC (*P* < 0.0001). 4/143 (3%) had completion thyroidectomies due to growth of preexisting nodules; no patient had a recurrence.

**Conclusion:**

One-third of differentiated thyroid cancer patients require levothyroxine treatment 3 years after hemithyroidectomy if postoperative thyroid-stimulating hormone levels up to 4 mIU/L are accepted. Avoidance of levothyroxine treatment happens at the expense of a significant increase in thyroid-stimulating hormone levels.

## Introduction

Hemithyroidectomy (HT) has recently become an eligible surgical treatment for a larger patient group with differentiated thyroid cancer (DTC) with low risk of recurrence. The revised American Thyroid Association’s (ATA) 2015 management guidelines for adult patients with thyroid nodules and DTC permit the treatment team and the patient to choose HT instead of total thyroidectomy (TT) for intrathyroidal papillary and follicular thyroid cancers of 1–4 cm without extrathyroidal extension or lymph node metastases that do not require radioiodine therapy (1).

Concerning thyroid function, HT has the great advantage over TT of potentially sparing some patients the need for daily postoperative thyroid hormone substitution therapy with levothyroxine (LT4). However, recent studies have demonstrated that the potential benefit of HT over TT in regard to postoperative thyroid function is much lower than first expected. In fact, adherence to the 2015 ATA guidelines to keep postoperative TSH below 2 mIU/L resulted in as many as 68–73% of low-risk DTC patients on LT4 after a year (2, 3).

Whereas the beneficial effect of TSH suppression on recurrence and survival is well known in intermediate- and high-risk DTC patients treated with TT and adjuvant radioactive iodine (4, 5, 6), recent studies have not supported the assumed association between TSH and recurrence in the low-risk hemithyroidectomized patient group (7, 8, 9).

However, most guidelines for the management of low-risk DTC recommend maintaining TSH in the low-normal range from 0.5 to 2 mIU/L after HT (1), and to routinely initiate LT4 treatment when postoperative TSH levels increase above 2 mIU/L to prevent TSH-mediated growth and development of nodules in the remnant lobe. Consequently, little is known about the prevalence of endogenous normal thyroid function in DTC patients when the compensatory TSH increase exceeds 2 mIU/L.

The Danish National Thyroid Cancer Guideline allows post-HT TSH to increase up to 4 mIU/L after HT for low-risk DTC before initiation of LT4 is recommended (10). Consequently, we have the unique possibility to examine the development of thyroid function changes after HT for DTC even after TSH increases above 2 mIU/L in a European patient cohort with previous iodine deficiency (11).

The trend toward more conservative treatment of DTC draws attention to the clinical importance of thyroid function changes after HT, and the hypothesis of this study states that by allowing post-HT TSH to increase up to 4 mIU/L, many patients will maintain endogenous normal thyroid function after HT for low-risk DTC. The aim of this study is to examine the prevalence of endogenous normal thyroid function in a cohort of DTC patients 3 years after HT for low-risk DTC.

## Materials and methods

### Patients

**Figure 1:**
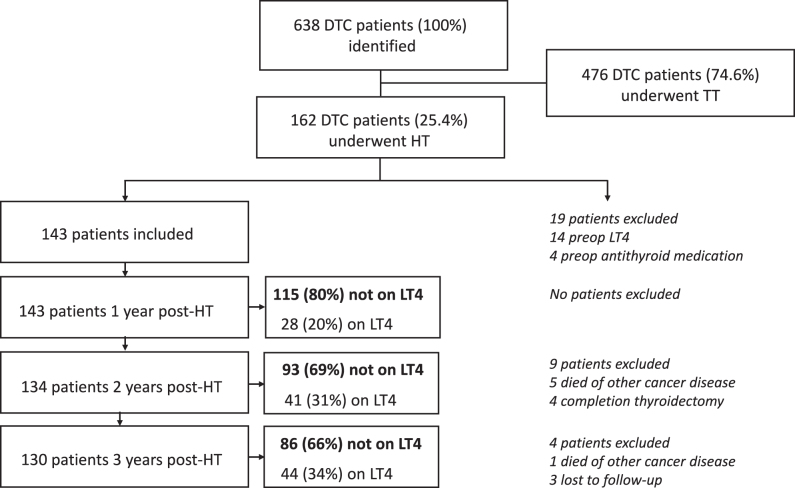
Study flow diagram of a 3-year follow-up of 162 Danish patients treated with hemithyroidectomy for DTC between July 2016 and July 2020. DTC, differentiated thyroid cancer; HT, hemithyroidectomy; TT, total thyroidectomy; LT4, levothyroxine.

This is a retrospective study of 162 DTC patients from Eastern Denmark treated with HT for DTC at three surgical departments between July 1st, 2016, and July 1st, 2020 (Fig. 1). We identified DTC patients in the national Danish Thyroid Cancer Database (DATHYRCA), a prospectively managed database including all Danish patients with a diagnosis of thyroid cancer (12). We obtained permission from the ethics committee to access patients’ hospital charts in a limited cohort of patients treated in Eastern Denmark (2.8 million inhabitants) with a diagnosis of DTC. In the 4-year study period, a total of 638 patients with surgically treated DTC were registered. 476/638 DTC patients (74.6%) underwent TT and 162/638 patients (25.4%) underwent initial HT and no completion thyroidectomy (Fig. 1). During the study period, the Danish national treatment guideline for DTC recommended HT only for tumors less than 2 cm, without extrathyroidal extension or lymph node metastasis. In the study population, only two patients had a tumor size above 2 cm (2.2 and 2.1 cm). In 2022, the Danish guideline was revised, and DTC patients with intrathyroidal tumors up to 4 cm are now offered HT, in accordance with the 2015 ATA guideline. After HT, the patients were referred to the oncological department at Herlev University Hospital, Capital Region, Denmark, for post-HT follow-up and control of thyroid function.

We reviewed patients’ hospital charts, medication lists, and pathology reports and documented postoperative LT4 treatment, along with pre- and postoperative TSH and disease recurrence. The inclusion criteria for the study were as follows: the patient underwent HT; the patient had been diagnosed with DTC; preoperative TSH level within the laboratory reference range (0.4–4.8 mIU/L); the patients were on no drugs known to affect thyroid function or thyroid hormone metabolism, such as a steroid, amiodarone, lithium, and beta-blocker. Patients were excluded from the study if they received preoperative thyroid hormone supplementation (15) or antithyroid medication (4). Figure 1 shows a flow study diagram of patients from the DATHYRCA database (12) who underwent HT for DTC and were included in the study. As demonstrated in the figure, 143/162 (88%) DTC patients who did not take thyroid hormone substitution before HT were ultimately included in the study.

### Surgical treatment

During the study period, the Danish national guideline recommended HT only for tumors less than 2 cm (10). Hemithyroidectomy with or without dissection of the ipsilateral level 6 of the neck was performed in an inpatient setting by experienced thyroid surgeons who all used an identical technique, which involved total left or right thyroid lobectomy and isthmectomy and, if present, resection of the pyramidal lobe with preservation of the contralateral thyroid lobe.

### Thyroid function

Measurement of serum TSH was performed with a third-generation electrochemical luminescent immunoassay. The reference range for TSH was 0.4–4.8 mIU/L.

Measurement of FT4 was performed with a chemiluminescent immunoassay. The reference range for FT4 was 11.8–24.0 pmol/L.

Measurement of the cancer biomarker thyroglobulin (Tg, ng/mL) was performed with a high-sensitivity immunoassay. The expected range of Tg in hemithyroidectomized patients is 1–20 ng/mL, depending on the remnant lobe.

### Postoperative follow-up

According to the Danish national guideline for management of low-risk DTC, post-HT TSH increase up to 4 mIU/L is accepted, and patients were initially followed up without LT4 (10). The first scheduled follow-up consultation was 1 month after surgery. In the first postoperative year, patients were scheduled for visits every 3–6 months, and thereafter, patients were scheduled for an annual visit for 2 more years. At each consultation, the patients underwent a medical examination, including medical history, current medication, and physical examination, and a non-fasting venous blood sample was drawn and submitted to analysis. LT4 treatment was initiated if a patient developed a TSH increase of >4 mIU/L or had hypothyroid symptoms, and doses were administered and adjusted to maintain the patient’s serum TSH values below 4 mIU/L. Ultrasonography examination of the remnant thyroid lobe and the neck was performed at 6 and 12 months postoperatively, and yearly hereafter. After a 3-year follow-up at the oncological department, patients were referred to their local department of endocrinology. During the 3 years of follow-up, no patients experienced a recurrence of DTC.

### Ethical considerations

The study was approved by the ethics committee of the Capital Region of Denmark (R-22032551), and permission to access the patients’ hospital charts was obtained. In addition, approval from the Danish Data Protection Agency was received. The study conformed to the principles of the Declaration of Helsinki.

### Statistical analysis

Statistical comparisons of categorical variables and continuous variables between ever and never users were performed by the Chi-square test and Wilcoxon rank-sum test, respectively, in Table 1.

**Table 1:** Baseline pre-operative characteristics of 143 patients before hemithyroidectomy for low-risk DTC. Values are presented as median and interquartile ranges. Mann–Whitney U test was used to analyze statistical significance (never vs ever on LT4).

Characteristics	All		Never LT4		Ever LT4		*P* values
	*n*	Values	*n*	Values	*n*	Values	
Age, years	143	53 (43–65)	99	53 (43–65)	44	51 (43.5–64)	0.73
Females, *n* (%)	143	115 (80)		77 (78)		38 (86)	0.23
BMI	143	26.0 (22.8–29.4)	99	26.0 (23.3–29.1)	44	25.3 (21.7–30.7)	0.78
Pre-HT TSH (U/mL)	116	1.29 (0.81–1.91)	78	1.12 (0.73–1.61)	38	1.89 (1.28–2.64)	<0.0001
TSH, mIU/L							
<2	90	1.10 (0.73–1.50)	70	1.04 (0.72–1.43)	20	1.28 (0.87–1.58)	0.004
2–4	26	2.62 (2.33–2.98)	8	2.49 (2.30–2.80)	18	2.72 (2.45–3.29)	0.004
>4	0	-	0	-		-	
FT4							
pmol/L	59	15.3 (13.7–17.5)	37	15.5 (14.9–18.0)	22	14.7 (13.6–16.1)	0.08
ng/dL	59	1.19 (1.06–1.36)	37	1.20 (1.16–1.40)	22	1.14 (1.06–1.25)	0.08

LT4, levothyroxine; BMI, body mass index; TSH, thyroid-stimulating hormone; FT4, free thyroxine; DTC, differentiated thyroid cancer.

As the data were retrieved from hospital-based data, the patients constituted an open dynamic cohort, where treatment status could change over time and patients could be censored due to death, loss to follow-up, or TT; thus, the open cohort reflects the clinician’s perspective at each time point moving forward. In this open cohort design, pairwise statistical comparisons of TSH preoperatively and at the different time points of follow-up were performed by the Wilcoxon signed-rank test, but this requires balanced data; thus, pairwise comparisons could only be done in patients who had both pre- and postoperative TSH values.

In a retrospective design, we also analyzed the repeated measurements of TSH, FT4, and Tg. We fitted a mixed-effects model to assess how thyroid function (TSH, FT4) and the thyroid cancer biomarker thyroglobulin changed over time for LT4 treatment initiated early (0–12 months), intermediate (12–24 months), or late (24–36 months) postoperatively. The models included fixed effects for LT4, time, and their interaction, plus covariates (age, sex). Random intercepts and slopes for time were included at the individual level (id). Maximum likelihood estimation was used to assess both preoperative differences and time trends, accounting for within- and between-subject variability. Covariance structure was not included, as assumptions about this did not change the model fit significantly. Neither age nor sex had a significant effect in the models. The mixed-effects model does not need balanced data (i.e., the same number of observations per time point), and therefore uses all available data for each time point, as compared to paired comparisons at each time point, which need balanced data. Therefore, results using the open dynamic cohort design and the retrospective repeated measures analyses may vary slightly.

A significant difference was defined as *P* < 0.05. The STATA statistical package software program (version 12.0; StataCorp, USA) was used for statistical analyses.

## Results

### Preoperative characteristics

A total of 143 patients were included, and all attended the 1-year follow-up consultation. Of these, thirteen patients were excluded during the follow-up period (Fig. 1): in the second year, five patients died of other cancer diseases, and four had completion thyroidectomies due to growth of preexisting nodules of the contralateral lobe and compressive symptoms, leaving 134 patients. Histology revealed benign multinodular goiter. During the third year, an additional three were lost to follow-up, and one patient died of another cancer disease, leaving 130 patients. The median follow-up was 36 months (IQR: 36–36).

The preoperative median TSH was 1.29 mUI/L (interquartile range (IQR): 0.81–1.91 mIU/L) in 116/143 patients (81%) (Table 1), but missing in 27/143 patients (19%). Patients who were never started on LT4 (never LT4: *n* = 99) during a 3-year follow-up after HT had a lower preoperative TSH (median (IQR): 1.12 mUI/L (0.73–1.61 mUI/L)) compared to patients who were started on LT4 treatment (ever LT4: *n* = 44) (1.89 mUI/L (1.28–2.64 mUI/L)) (*P* < 0.0001) (Table 1). The median age was 53 years (IQR: 43–65 years), and 115/143 (80%) were women. Age, sex, and BMI were not different between the groups preoperatively.

### Differentiated thyroid cancer pathology

Histology revealed papillary thyroid carcinoma (PTC) in 137/143, and follicular thyroid carcinoma (FTC) in 6/143 patients (Table 2). In 141/143 patients, the tumor was less than 2 cm, and 2/143 patients had tumors of 2.2 and 2.1 cm. The median tumor diameter was 0.8 cm (IQR: 0.3–1.3 cm). All tumors were intrathyroidal with neither minimal nor gross extrathyroidal extension. None of the patients had nodal metastasis. All were classified as low risk of recurrence based on the American Thyroid Association risk stratification system (1).

**Table 2:** Indication for hemithyroidectomy and postoperative pathology results.

Characteristics	Values
Total, *n*	143
Incidental focal lesion, *n* (%)	46 (32)
PET-CT, *n*	33
Neck-CT, *n*	7
Neck-MRI, *n*	4
Neck-US, *n*	2
Goiter or nodule, *n* (%)	97 (68)
Compressive, *n*	33
Cosmetic, *n*	8
PTC, *n* (%)	137 (96)
Incidental, *n* (% of PTC)*	60 (44)
Tumor size, mm	3 (2.0–4.5)
Suspected malign nodule, *n* (% of PTC)	83 (66%)
Tumor size, mm	12 (9–17)
Multifocal disease, *n* (% of PTC)	6 (5)
FTC, *n* (%)	6 (4)
Concomitant benign nodular goiter, *n* (%)	85 (59)
Concomitant lymphocytic thyroiditis, *n* (%)	11 (8)
Tumor size, mm	8 (3–13)

* Incidental findings during routine pathologic examination after HT for goiter.

PET-CT, positron emission tomography–computed tomography; MRI, magnetic resonance imaging; US, ultrasound scan; DTC, differentiated thyroid cancer; PTC, papillary thyroid cancer; FTC, follicular thyroid cancer.

### Thyroid function 1, 2, and 3 years after hemithyroidectomy

During a 3-year follow-up, the prevalence of endogenous normal thyroid function (i.e. TSH: 0.4–4.8 mIU/L) gradually decreased to 80, 69, and 66% after 1, 2, and 3 years (Table 3). This was followed by a concomitant development of hypothyroidism with TSH >4.0 mIU/L in 20, 31, and 34% of patients, who were replaced with LT4 after 1, 2, and 3 years.

**Table 3:** Serum TSH levels before and after hemithyroidectomy for DTC during a 3-year follow-up stratified by levothyroxine treatment in the open dynamic cohort design. Values indicate median and interquartile ranges. Wilcoxon signed-rank test was used to analyze statistical significance (pre- and post-hemithyroidectomy).

Patient groups	1-year post HT (*n* = 143)		2-year post HT (*n* = 134)*		3-year post HT (*n* = 130)^†^	
	Complete	Missing	Complete	Missing	Complete	Missing
HT +/− LT4						
TSH pre/post complete, *n*	116	27	106	28	96	34
Pre-HT TSH mIU/L	1.29 (0.81–1.91)	-	1.30 (0.82–1.92)	-	1.3 (0.78–1.93)	-
Post-HT TSH mIU/L	2.23 (1.71–3.16)	2.4 (1.70–3.16)	2.22 (1.58–3.06)	2.29 (1.69–2.81)	2.1 (1.45–2.87)	1.91 (1.49–2.71)
*P*-value	<0.0001	-	<0.0001	-	<0.0001	-
HT without LT4						
TSH pre/post complete, *n*	93	22	73	20	63	23
Pre-HT TSH mIU/L	1.16 (0.74–1.64)	-	1.20 (0.74–1.64)	-	1.16 (0.79–1.70)	-
Post-HT TSH mIU/L	2.15 (1.71–3.0)	2.35 (1.69–3.16)	2.26 (1.70–3.06)	1.96 (1.22–2.39)	1.94 (1.38–2.65)	1.92 (1.50–2.50)
*P*-value	<0.0001	-	<0.0001	-	<0.0001	-
TSH 2–4 mIU/L, *n* (%)	68 (59)		46 (43)		36 (42)	
HT with LT4						
TSH pre/post complete, *n*	23	5	36	5	39	5
Pre-HT TSH mIU/L	2.45 (1.4–3.21)	-	1.98 (1.28–2.82)	-	1.98 (1.28–2.82)	-
TSH at LT4 initiation, mIU/L	6.25 (5.31–9.96)		5.80 (5.20–8.73)		5.88 (5.20–6.18)	
Post-HT TSH mIU/L	3.01 (1.61–4.80)	2.40 (2.00–2.42)	2.13 (1.49–3.11)	2.61 (2.45–4.84)	2.48 (1.46–3.22)	1.6 (1.34–1.92)
*P*-value for pre vs post TSH	0.017	-	0.14	-	0.13	-

HT, hemithyroidectomy; TSH, thyroid-stimulating hormone; LT4; levothyroxine; DTC, differentiated thyroid cancer.

* 9 patients were excluded between the 1- and 2-year follow-up.

^†^ 4 patients were excluded between the 2- and 3-year follow-up.

Table 3 demonstrates pre- and post-hemithyroidectomy serum levels of TSH during a 3-year follow-up stratified by LT4 treatment. In the patient group with maintained endogenous normal thyroid function, and therefore not on LT4 during the 3-year follow-up, TSH was significantly increased within the normal range 1, 2, and 3 years after hemithyroidectomy for DTC (*P* < 0.0001). In addition, patients on LT4 1 year after HT for DTC had an increased TSH compared to preoperative values, but 2 and 3 years after HT, TSH did not differ from preoperative values in this patient group (Table 3). The median TSH values upon initiation of LT4 supplementation during the 3-year follow-up are also demonstrated in Table 3.

### Thyroid function and the need for LT4 according to the ATA guidelines

One year post-HT, 28/143 (20%) patients had developed hypothyroidism with TSH >4 mIU/L, and 68/143 (48%) patients had a TSH increase from 2 to 4 mIU/L. 2 years after HT, the incidence of LT4 treatment had increased to 41/134 (31%), and 46/134 (34%) patients had increased TSH to between 2 and 4 mIU/L. Finally, 44/130 (34%) hemithyroidectomized patients were on LT4 after 3 years, and 36/130 (28%) patients had a TSH increase from 2 to 4 mIU/L.

**Figure 2:**
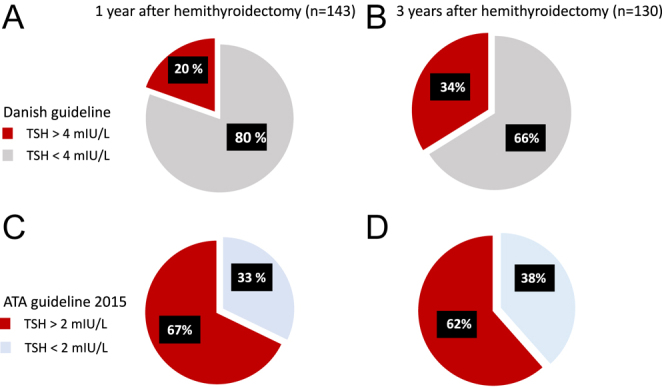
(A, B, C, D) Diagrams demonstrating the prevalence of levothyroxine treatment according to the postoperative TSH goal 1 and 3 years after hemithyroidectomy for DTC. TSH, thyroid stimulating hormone; ATA, American Thyroid Association.

If we look at the data with the intention to adhere to the 2015 ATA guidelines that recommend maintaining post-HT TSH below 2 mIU/L, we find that 28 + 68 = 96 of 143 (67%) would have been on LT4 1 year after HT. 2 years after HT for DTC, the number of patients with an indication for LT4 treatment according to the 2015 ATA guidelines would be 41 + 46 = 87 of 134 (65%), and after a 3-year follow-up, adherence to the 2015 ATA guidelines would result in 44 + 36 = 80 of 130 (62%) patients on LT4 after HT for DTC. The prevalences of LT4 treatment 1 and 3 years after HT for DTC according to the post-HT TSH goal are presented in Fig. 2.

### Remnant thyroid lobe ultrasound characteristics

Ultrasonography (US) of the contralateral thyroid lobe and the neck was performed 6 and 12 months after HT and yearly thereafter during the 3-year follow-up. The ultrasound reports focused on the detection of DTC recurrence and included information on neck lymph nodes and on the nodularity, but not on the volume of the remnant lobe. The first postoperative US demonstrated that 96/143 (67%) patients treated with HT for DTC had either one (21/143) or multiple nodules (75/143) in the remnant thyroid lobe. All were described as non-suspicious of cancer. Of the patients, 47/143 (33%) had no nodules in the remnant thyroid lobe. During follow-up, 19/143 (13%) patients experienced growth of nodules (>3 mm in one diameter), and 4/143 (3%) had completion thyroidectomies due to compression symptoms; histology revealed benign nodular goiter. The appearance of new nodules during the follow-up was described in 10/47 (21%) patients.

### Repeated measurements of TSH, FT4, and thyroglobulin

**Figure 3:**
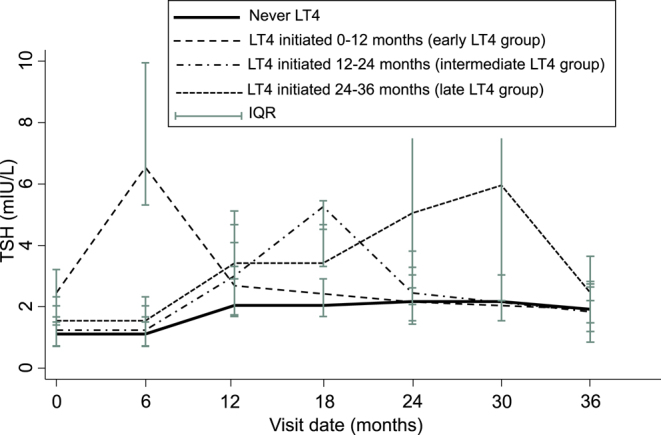
Repeated measurements of TSH (mIU/L) according to levothyroxine (LT4) treatment after hemithyroidectomy for DTC. Values are presented as median (IQR).

TSH time trends differed across groups (*P* < 0.0001). TSH increased over time in the ‘never LT4’ group (*P* < 0.001) (Supplemental Tables 1 and 2 (see section on Supplementary materials given at the end of the article), Fig. 3). The early LT4 group had higher preoperative TSH, which also increased after hemithyroidectomy and then declined over time (*P* < 0.0001) (Supplemental Tables 1 and 2, Fig. 2). The intermediate and late LT4 groups had no significant TSH difference preoperatively and did not change significantly over time. While the preoperative TSH within each treatment group was homogeneous (variance = 0), there was heterogeneity in time trends, i.e., some patients responded differently over time even within the same treatment group (Supplemental Table 1). Most of the variance in TSH was unexplained within-person visit-to-visit variability.

FT4 time trends differed across groups (*P* < 0.0001) (Supplemental Tables 3 and 4a and b, Supplemental Fig. 1). FT4 declined over time in the ‘never LT4’ group (*P* = 0.001). FT4 did not differ preoperatively due to high variation in preoperative FT4 between individuals (Supplemental Table 3). The early and the intermediate LT4 groups showed increasing FT4 levels over time after treatment initiation (*P* = 0.001).

Thyroglobulin increased over time in the ‘never LT4’ group (*P* = 0.02) (Supplemental Tables 5 and 6). There was high between-person variability for each treatment group, and there was no significant evidence that thyroglobulin trends over time differed between the treatment groups overall (*P* = 0.1172).

## Discussion

Our study demonstrates that most low-risk DTC patients can avoid LT4 treatment after therapeutic HT if a postoperative TSH increase up to 4 mIU/L is accepted. In our Danish cohort, 80% of patients had maintained endogenous normal thyroid function 1 year after HT, and only 20% of DTC patients were on LT4. After 3 years, the prevalence of endogenous normal thyroid function was 66%, which means that only 34% of DTC patients were on LT4. The serial measurements of TSH and FT4 trends aligned with expected physiologic or treatment-driven compensation. No patient had a recurrence of DTC, but four patients had completion thyroidectomies performed due to growth and compressive symptoms of preexisting benign nodules of the contralateral thyroid lobe. If LT4 treatment in our cohort had been initiated when postoperative TSH increased above 2 mIU/L, 67% of DTC patients would have been on LT4 after the first year and 64% after 3 years. These results are in accordance with previous studies, in which adherence to the ATA guideline of postoperative management of TSH after HT for DTC resulted in as many as 68–78% of patients on LT4 after a year (2, 3, 13). The present study has clearly demonstrated that the possibility to avoid postoperative LT4 treatment in low-risk DTC patients is directly related to the recommended postoperative TSH goal. Consequently, thyroid function changes after HT, and the optimal postoperative TSH level in the growing population of hemithyroidectomized DTC patients becomes of paramount interest.

Following HT, the pituitary–thyroid axis adapts to the loss of thyroid tissue with a compensative TSH increase to allow the contralateral lobe to produce enough thyroid hormone to maintain euthyroidism. Failure of this compensation results in postoperative hypothyroidism, with a reported incidence ranging from 6 to 56% depending on the follow-up period and on the definition of hypothyroidism (14, 15, 16, 17). Notably, the thyroglobulin increase over time in the ‘never LT4’ group could suggest an increase in the volume or functional activity of the remnant lobe in hemithyroidectomized patients, but it is not a specific or reliable indicator of volume alone.

During the past three decades, the incidence of DTC in Denmark and worldwide has steadily increased, and worldwide, DTC is now the most common endocrine cancer (18, 19, 20), probably due to widespread use of high-resolution ultrasonography and other imaging modalities resulting in the detection of clinically occult thyroid cancers (21). The overdiagnosis of DTC has become a worldwide health problem (22).

Due to the indolent nature of DTC, balancing treatment risks with risks of disease progression is challenging. The present ATA recommendation to maintain post-HT TSH in the mid to lower reference range (0.5–2.0 mIU/L) reflects an extrapolation of the beneficial effect on recurrence and survival known from intermediate- and high-risk patients after TT to low-risk patients after HT (23). At present, the association between TSH and recurrence after HT in low-risk DTC patients is unclear. In one study, serum TSH >1.85 mIU/L independently predicted a higher risk of recurrence within the first 2 years after HT in patients with low- or intermediate-risk PTC after HT (24). However, several other studies have not supported the presumption that TSH suppression has a beneficial effect on the short-term recurrence of low-risk DTC and have concluded that a TSH increase above 2 mIU/L after HT has no impact on oncological safety (7, 8, 9). Another consideration after HT for DTC is the growth of nodules and the development of new nodules of the remnant lobe. Recently, the 20-year follow-up data of a randomized controlled trial demonstrated that prophylactic LT4 treatment significantly decreased the goiter recurrence rate and the need for completion thyroidectomy among iodine-deficient patients (25). In populations such as the Danish, with previous iodine deficiency and high prevalence of multinodular goiter (11), LT4 treatment after HT for DTC might not be necessary to decrease the risk of cancer recurrence but to prevent the growth of preexisting and new nodules of the contralateral lobe. In 2022, the revised Danish national treatment guideline widened the indications for HT in concordance with the 2015 ATA guideline (1), and tumors up to 4 cm are presently treated with HT. In the study cohort, the majority had contralateral nodules, and the fact that 4 out of 143 (3%) in the study cohort had completion thyroidectomies during the 3-year follow-up calls attention to the need for more research about post-HT TSH maintenance in this patient group and the advantages and disadvantages of introducing HT as a treatment option for DTC in a previously iodine-deficient European population.

In this study, we have demonstrated that by allowing post-HT TSH to increase up to 4 mIU/L, most patients can maintain their endogenous normal thyroid function after HT for low-risk DTC and are not on LT4 3 years after HT. Postoperative lifelong daily medication with LT4 treatment and regular testing of thyroid function are inevitable consequences of TT, but also for the many patients who start LT4 treatment after HT following the ATA guideline for TSH management. Acceptance of TSH increase up to 4 mIU/L reduces the need for LT4 treatment, which may enhance the benefits of HT over TT as a treatment option of DTC but happens at the expense of a significant postoperative increase in TSH.

In addition, HT has other benefits over TT than the possibility to avoid lifelong LT4 treatment. Unilateral thyroid surgery places the recurrent laryngeal nerve at a lower risk of injury, and moreover, it eliminates the risk of bilateral injury to the recurrent laryngeal nerves (26) and postoperative hypoparathyroidism (27), both of which result in deficits in quality of life.

Our study has several possible limitations. First, information on TSH, FT4, and thyroglobulin was incomplete due to the variability in documentation and data capture in routine care settings. This limitation can impact repeated measures analyses by reducing statistical power and potentially introducing bias if the missing data are not random or systematically related to patient characteristics or outcomes. In addition, we lacked data on well-known risk factors for postoperative hypothyroidism, such as serum TPO antibody status. Second, the postoperative ultrasound examination reports focused on DTC recurrence and contained only information on neck lymph nodes and nodularity of the remnant thyroid lobe but did not include information on remnant lobe volume. In addition, the study is limited by the relatively short duration of follow-up, and it is possible that with longer follow-up, more patients might experience recurrences of DTC and goiter. A major strength of the study is the cohort of 143 patients with clinical data and the symptoms of thyroid nodular disease leading to the indication for surgery and the diagnosis of low-risk DTC.

In conclusion, 66% of patients had maintained endogenous normal thyroid function 3 years after HT for DTC and were not on LT4 treatment, which demonstrates that most low-risk DTC patients can avoid postoperative LT4 treatment if TSH is allowed to increase up to 4 mIU/L. Over the last decade, HT has become an eligible surgical treatment for a larger DTC patient group, and acceptance of TSH increase to the high normal reference range greatly reduces the need for LT4 treatment and may be considered as a benefit of HT over TT in the treatment of DTC. However, the avoidance of LT4 treatment happens at the expense of a significant increase in postoperative TSH. The thyroid function of each individual is unique (28), and a significant change in TSH within the reference range after HT may represent an abnormal thyroid function and has been associated with weight gain (29) and decreased mitochondrial function (17), indicating a state of individual subclinical hypothyroidism. We call attention to the possible long-term biological consequences of a change in the individual TSH set point after HT for DTC, such as goiter recurrence and a possible risk of cardiovascular disease, and suggest that LT4 treatment after HT for DTC is initiated in consideration of the preoperative TSH set point of the individual. Future research in the optimal postoperative TSH level of the individual in the growing population of hemithyroidectomized DTC patients is warranted.

## Supplementary materials



## Declaration of interest

The authors declare no conflicts of interest that could be perceived as prejudicing the impartiality of the study reported.

## Funding

The research project did not receive any specific grant from any funding agency in the public, commercial, or not-for-profit sector.

## Author contribution statement

TTK designed the study, and TTK and CE analyzed the data and wrote the manuscript. TTK led the data collection group (CCP, MATK, and JB), and all authors took part in the review and editing process of the present manuscript.

## References

[1] Haugen BR, Alexander EK, Bible KC, et al. 2015 American Thyroid Association Management Guidelines for adult patients with thyroid nodules and differentiated thyroid cancer: the American Thyroid Association Guidelines task force on thyroid nodules and differentiated thyroid cancer. Thyroid 2016 26 1–133. (10.1089/thy.2015.0020) 26462967 PMC4739132

[2] Cox C, Bosley M, Southerland LB, et al. Lobectomy for treatment of differentiated thyroid cancer: can patients avoid postoperative thyroid hormone supplementation and be compliant with the American Thyroid Association Guidelines? Surgery 2018 163 75–80. (10.1016/j.surg.2017.04.039) 29122328

[3] Schumm MA, Lechner MG, Shu ML, et al. Frequency of thyroid hormone replacement after lobectomy for differentiated thyroid cancer. Endocr Pract 2021 27 691–697. (10.1016/j.eprac.2021.01.004) 33642257

[4] Ruel E, Thomas S, Dinan M, et al. Adjuvant radioactive iodine therapy is associated with improved survival for patients with intermediate-risk papillary thyroid cancer. J Clin Endocrinol Metab 2015 100 1529–1536. (10.1210/jc.2014-4332) 25642591 PMC4399282

[5] Kazaure HS, Roman SA & Sosa JA. Aggressive variants of papillary thyroid cancer: incidence, characteristics and predictors of survival among 43,738 patients. Ann Surg Oncol 2012 19 1874–1880. (10.1245/s10434-011-2129-x) 22065195

[6] Kazaure HS, Roman SA & Sosa JA. Insular thyroid cancer: a population-level analysis of patient characteristics and predictors of survival. Cancer 2012 118 3260–3267. (10.1002/cncr.26638) 22252610

[7] Lee MC, Kim MJ, Choi HS, et al. Postoperative thyroid-stimulating hormone levels did not affect recurrence after thyroid lobectomy in patients with papillary thyroid cancer. Endocrinol Metab 2019 34 150–157. (10.3803/enm.2019.34.2.150) PMC6599911 31099202

[8] Ahn D, Lee GJ, Sohn JH, et al. Oncological impact of hypothyroidism and levothyroxine supplementation following hemithyroidectomy in patients with papillary thyroid carcinoma. Head Neck 2020 42 1004–1013. (10.1002/hed.26075) 31930773

[9] Xu S, Huang Y, Huang H, et al. Optimal serum thyrotropin level for patients with papillary thyroid carcinoma after lobectomy. Thyroid 2022 32 138–144. (10.1089/thy.2021.0404) 34617446

[10] Danish Thyroid Cancer GD. Danish national thyroid cancer treatment guideline. 2022. (www.dahanca.dk)

[11] Tang Møllehave L, Knudsen N, Linneberg A, et al. The Danish investigation on iodine intake and thyroid disease (DanThyr): history and implications. Eur Thyroid J 2024 13 e230230. (10.1530/etj-23-0230) 38657651 PMC11227094

[12] Londero SC, Mathiesen JS, Krogdahl A, et al. Completeness and validity in a national clinical thyroid cancer database: DATHYRCA. Cancer Epidemiol 2014 38 633–637. (10.1016/j.canep.2014.07.009) 25132423

[13] Wilson M, Patel A, Goldner W, et al. Postoperative thyroid hormone supplementation rates following thyroid lobectomy. Am J Surg 2021 221 804–808. (10.1016/j.amjsurg.2020.07.001) 32682499

[14] Verloop H, Louwerens M, Schoones JW, et al. Risk of hypothyroidism following hemithyroidectomy: systematic review and meta-analysis of prognostic studies. J Clin Endocrinol Metab 2012 97 2243–2255. (10.1210/jc.2012-1063) 22511795

[15] Ahn D, Sohn JH & Jeon JH. Hypothyroidism following hemithyroidectomy: incidence, risk factors, and clinical characteristics. J Clin Endocrinol Metab 2016 101 1429–1436. (10.1210/jc.2015-3997) 26900643

[16] Park S, Jeon MJ, Song E, et al. Clinical features of early and late postoperative hypothyroidism after lobectomy. J Clin Endocrinol Metab 2017 102 1317–1324. (10.1210/jc.2016-3597) 28324106

[17] Toft Kristensen T, Larsen J, Pedersen PL, et al. Persistent cellular metabolic changes after hemithyroidectomy for benign euthyroid goiter. Eur Thyroid J 2014 3 10–16. (10.1159/000357943) 24847460 PMC4005256

[18] Pellegriti G, Frasca F, Regalbuto C, et al. Worldwide increasing incidence of thyroid cancer: update on epidemiology and risk factors. J Cancer Epidemiol 2013 2013 965212. (10.1155/2013/965212) 23737785 PMC3664492

[19] Deng Y, Li H, Wang M, et al. Global burden of thyroid cancer from 1990 to 2017. JAMA Netw Open 2020 3 e208759. (10.1001/jamanetworkopen.2020.8759) 32589231 PMC7320301

[20] Sorensen SM, de la Cour CD, Maltesen T, et al. Temporal trends in papillary and follicular thyroid cancer incidence from 1995 to 2019 in adults in Denmark according to education and income. Thyroid 2022 32 972–982. (10.1089/thy.2021.0602) 35459415

[21] Wiltshire JJ, Drake TM, Uttley L, et al. Systematic review of trends in the incidence rates of thyroid cancer. Thyroid 2016 26 1541–1552. (10.1089/thy.2016.0100) 27571228

[22] Pizzato M, Li M, Vignat J, et al. The epidemiological landscape of thyroid cancer worldwide: GLOBOCAN estimates for incidence and mortality rates in 2020. Lancet Diabetes Endocrinol 2022 10 264–272. (10.1016/s2213-8587(22)00035-3) 35271818

[23] Biondi B & Cooper DS. Benefits of thyrotropin suppression versus the risks of adverse effects in differentiated thyroid cancer. Thyroid 2010 20 135–146. (10.1089/thy.2009.0311) 20151821

[24] Park JH, Lee YM, Lee YH, et al. The prognostic value of serum thyroid-stimulating hormone level post-lobectomy in low- and intermediate-risk papillary thyroid carcinoma. J Surg Oncol 2018 118 390–396. (10.1002/jso.25164) 30114333

[25] Barczynski M, Golkowski F, Hubalewska-Dydejczyk A, et al. Twenty-year follow-up of a randomized clinical trial of unilateral thyroid lobectomy with or without postoperative levothyroxine treatment. World J Surg 2025 49 140–147. (10.1002/wjs.12403) 39547954

[26] Schneider R, Randolph GW, Dionigi G, et al. International neural monitoring study group guideline 2018 part I: staging bilateral thyroid surgery with monitoring loss of signal. Laryngoscope 2018 128 (Supplement 3) S1–S17. (10.1002/lary.27359) 30289983

[27] Anneback M, Hedberg J, Almquist M, et al. Risk of permanent hypoparathyroidism after total thyroidectomy for benign disease: a nationwide population-based cohort study from Sweden. Ann Surg 2021 274 e1202–e1208. (10.1097/sla.0000000000003800) 32032086

[28] Andersen S, Pedersen KM, Bruun NH, et al. Narrow individual variations in serum T(4) and T(3) in normal subjects: a clue to the understanding of subclinical thyroid disease. J Clin Endocrinol Metab 2002 87 1068–1072. (10.1210/jc.87.3.1068) 11889165

[29] Toft Kristensen T, Larsen J, Pedersen PL, et al. Weight gain and serum TSH increase within the reference range after hemithyroidectomy indicate lowered thyroid function. J Thyroid Res 2014 2014 892573. (10.1155/2014/892573) 24959372 PMC4052094

