# Supplementary material for: Prevalence of endogenous normal thyroid function 3 years after hemithyroidectomy for differentiated thyroid cancer
Source: Eur Thyroid J. 2025 Sep 11;14(5):e240282. doi: 10.1530/ETJ-24-0282 (PMC12558410; doi:10.1530/ETJ-24-0282)

Supplement

Title: Prevalence of endogenous normal thyroid function three years after hemithyroidectomy for differentiated thyroid cancer

Authors: Tina Toft Kristensen, Christina Caroline Plaschke, Anne Fog Lomholt, Christoffer Holst Hahn, Irene Wessel, Mads Aage Toft Kristensen, Jens Bentzen, Christina Ellervik

Supplemental Table 1. Mixed effects models of TSH.

Legend:

Model: mixed TSH i.eltroxcat##c.visit_date age sex || id:visit_date , mle

TSH: thyroid-stimulating hormone (mIU/L). Eltroxcat (0: never treated, 1: LT4 initiated 0-12 months, 2:LT4 initiated 12-24 months, 3: LT4 initiated 24-36 months). Visit_date (0, 6, 12, 18, 24, 30, 36 months). Age (years, continuous). Sex (0: female, 1: male). MLE: maximum likelihood estimation.

**TSH (mIU/L)**				
**Term**	**Coef (β)**	**SE**	**P-value**	**Meaning**
eltroxcat 1 (early treatment group)	3.604	0.331	0.0000	Pre-operatively, TSH is much higher than in the reference group (eltroxcat 0: no treatment)
eltroxcat 2 (intermediate treatment group)	0.779	0.443	0.0790	Higher pre-operatively, borderline significant
eltroxcat 3 (late treatment group)	0.968	0.831	0.2440	Pre-operatively, not statistically different than in the reference group (eltroxcat 0: no treatment)
visit_date	0.041	0.009	0.0000	TSH increases over time in the reference group (eltroxcat 0: no treatment)
eltroxcat 1 # visit_date	-0.121	0.019	0.0000	In the early eltroxin treatment group, slow TSH descrease over time after treatment initiation compared to reference group (eltroxcat 0: no treatment)
eltroxcat 2 # visit_date	-0.002	0.027	0.9470	No slope difference from reference group (eltroxcat 0: no treatment)
eltroxcat 3 # visit_date	0.017	0.048	0.7240	No slope difference from reference group (eltroxcat 0: no treatment)
Age	0.003	0.007	0.6380	Not significantly associated with TSH
Sex	-0.117	0.243	0.6290	Also not significantly associated with TSH
**Component**	**Variance**			**Interpretation**
var(_cons)	~0 (1.17e-13)			Practically no random intercept variation, i.e., no meaningful between-subject differences in pre-operative TSH after adjusting for fixed effects
var(visit_date)	0.002			Some variability in individual slopes over time
var(Residual)	4.240			Most variation is within-person, not between-person
Interaction	chi2(3) = 40.25, p < 0.0000			
LR test vs. linear model:	chi2(2) = 65.92		Prob > chi2 = 0.0000	

Supplemental Table 2. Repeated measurements of TSH (mIU/L).

LT4	Month	N	median	IQR25	IQR75
No	0	78	1.12	0.73	1.61
No	6	78	1.12	0.73	1.61
No	12	99	2.09	1.69	2.93
No	18	90	2.07	1.69	2.88
No	24	88	2.22	1.61	3.02
No	30	78	2.17	1.55	3.03
No	36	84	1.94	1.48	2.68
Early	0	23	2.45	1.40	3.21
Early	6	26	6.54	5.31	9.95
Early	12	28	2.69	1.72	4.09
Early	24	27	2.16	1.43	3.81
Early	36	25	1.92	1.20	2.82
Intermediate	0	12	1.24	0.71	2.03
Intermediate	6	12	1.24	0.71	2.03
Intermediate	12	13	3.00	1.74	5.12
Intermediate	18	10	5.26	4.52	5.46
Intermediate	24	10	2.45	2.08	3.28
Intermediate	36	11	1.84	0.85	3.65
Late	0	3	1.55	1.50	2.33
Late	6	3	1.55	1.50	2.33
Late	12	3	3.43	3.31	4.67
Late	18	3	3.43	3.31	4.67
Late	24	2	5.05	2.61	7.49
Late	30	3	5.96	2.20	7.49
Late	36	3	2.46	2.20	2.74

Supplemental Table 3. Mixed effects models of FT4 (pmol/L).

Legend:

Model: mixed FT4 i.eltroxcat##c.visit_date age sex || id:visit_date , mle

FT4: free thyroxine (pmol/L). Eltroxcat (0: never treated, 1: LT4 initiated 0-12 months, 2:LT4 initiated 12-24 months, 3: LT4 initiated 24-36 months). Visit_date (0, 6, 12, 18, 24, 30, 36 months). Age (years, continuous). Sex (0: female, 1: male). MLE: maximum likelihood estimation.

**Free T4 (pmol/L)**				
**Variable**	**Coef (β)**		**p-value**	**Interpretation**
eltroxcat 1 (early treatment group)	-2.284	1.230	0.0630	Pre-operatively, the early treatment group has slightly lower FT4 than the reference group (eltroxcat 0: no treatment), borderline significant.
eltroxcat 2 (intermediate treatment group)	-1.332	1.681	0.4280	Pre-operatively, not significant difference from the reference group (eltroxcat 0: no treatment)
eltroxcat 3 (late treatment group)	-0.956	3.221	0.7670	Pre-operatively, not significant difference from the reference group (eltroxcat 0: no treatment)
visit_date	-0.030	0.009	0.0010	FT4 decreases over time for the reference group (eltroxcat 0: no treatment) — about 0.03 units per visit unit.
eltroxcat 1 # visit_date	0.153	0.019	<0.001	FT4 increases over time in the early treatment group, significantly different from the reference group's (eltroxcat 0: no treatment) decline.
eltroxcat 2 # visit_date	0.089	0.027	0.0010	FT4 increases over time in the intermediate treatment group, significantly different from the reference group's (eltroxcat 0: no treatment) decline.
eltroxcat 3 # visit_date	0.029	0.039	0.4600	No significant change in slope compared to the reference group (eltroxcat 0: no treatment).
Age	0.035	0.034	0.3050	Not significant. Age not strongly associated with FT4.
Sex	2.205	1.200	0.0660	Marginally significant — men may have higher FT4.
**Random Effect**	**Variance**			**Interpretation**
var(_cons)	28.680			High individual variation in pre-operative FT4 levels
var(visit_date)	0.003			Small variation in individual slopes over time (some people rise faster/slower)
var(Residual)	1.650			Measurement noise or within-person visit-to-visit fluctuation
Interaction	chi2(3) = 70.39 p < 0.0000			
LR test vs. linear model:	chi2(2) = 784.88		Prob > chi2 = 0.0000	

Supplemental Table 4a. Repeated measurements of FT4 (pmol/L).

LT4	Month	N	median	IQR25	IQR75
No	0	37	15.50	14.90	18.00
No	6	37	15.50	14.90	18.00
No	12	79	14.40	13.70	16.00
No	18	71	14.20	13.70	16.00
No	24	83	14.40	13.50	15.90
No	30	73	14.60	13.60	15.90
No	36	50	13.95	13.00	15.00
Early	0	13	14.40	13.70	15.70
Early	6	18	12.95	11.60	14.10
Early	12	20	15.75	14.25	17.70
Early	24	25	16.40	14.50	17.50
Early	36	16	18.05	16.05	20.30
Intermediate	0	6	14.30	13.20	17.50
Intermediate	6	6	14.30	13.20	17.50
Intermediate	12	12	14.80	13.25	15.95
Intermediate	18	8	15.20	14.00	16.35
Intermediate	24	11	16.70	16.00	18.40
Intermediate	36	7	14.40	13.00	19.00
Late	0	3	15.10	11.20	16.20
Late	6	3	15.10	11.20	16.20
Late	12	3	14.10	13.00	14.90
Late	18	3	14.10	13.00	14.90
Late	24	2	14.35	12.70	16.00
Late	30	1	12.70	12.70	12.70
Late	36	3	14.00	12.00	17.40

Supplemental Table 4b. Repeated measurements of FT4 (ng/dL).

LT4	Month	N	median	IQR25	IQR75
No	0	37	1.20	1.16	1.40
No	6	37	1.20	1.16	1.40
No	12	79	1.12	1.06	1.24
No	18	71	1.10	1.06	1.24
No	24	83	1.12	1.05	1.24
No	30	73	1.13	1.06	1.24
No	36	50	1.08	1.01	1.17
Early	0	13	1.12	1.06	1.22
Early	6	18	1.01	0.90	1.10
Early	12	20	1.22	1.11	1.38
Early	24	25	1.27	1.13	1.36
Early	36	16	1.40	1.25	1.58
Intermediate	0	6	1.11	1.03	1.36
Intermediate	6	6	1.11	1.03	1.36
Intermediate	12	12	1.15	1.03	1.24
Intermediate	18	8	1.18	1.09	1.27
Intermediate	24	11	1.30	1.24	1.43
Intermediate	36	7	1.12	1.01	1.48
Late	0	3	1.17	0.87	1.26
Late	6	3	1.17	0.87	1.26
Late	12	3	1.10	1.01	1.16
Late	18	3	1.10	1.01	1.16
Late	24	2	1.11	0.99	1.24
Late	30	1	0.99	0.99	0.99
Late	36	3	1.09	0.93	1.35

Supplemental Table 5. Mixed effects models of Thyroglobulin (ng/mL).

Legend:

Model: mixed Tg i.eltroxcat##c.visit_date age sex || id:visit_date , mle

Tg: Thyroglobulin (ng/mL). Eltroxcat (0: never treated, 1: LT4 initiated 0-12 months, 2:LT4 initiated 12-24 months, 3: LT4 initiated 24-36 months was not included due to too few N). Visit_date (0, 6, 12, 18, 24, 30, 36 months). Age (years, continuous). Sex (0: female, 1: male). MLE: maximum likelihood estimation.

**Thyroglobulin (ng/mL)**				
**Variable**	**Coef (β)**		**P-value**	**Interpretation**
eltroxcat 1	-3.855	5.141	0.453	Pre-operatively, not significant difference from the reference group (eltroxcat 0: no treatment)
eltroxcat 2	8.294	6.575	0.207	Pre-operatively, not significant difference from the reference group (eltroxcat 0: no treatment)
visit_date	0.289	0.122	0.018	Thyroglobulin increases over time in the reference group (eltroxcat 0: no treatment)
eltroxcat 1 # visit_date	0.117	0.263		No slope difference from the reference group (eltroxcat 0: no treatment)
eltroxcat 2 # visit_date	-0.702	0.364	0.054	Marginally significant decrease in slope compared to the reference group (eltroxcat 0: no treatment)
Age	0.012	0.084	0.888	No effect of age on thyroglobulin
Sex	-1.227	2.935	0.676	No significant association
**Random Effect**	**Variance**			**Interpretation**
var(_cons)	~0 (2.3e-15)			No between-subject variation in baseline thyroglobulin, after adjusting for fixed effects
var(visit_date)	0.323			Some variation in individual time slopes — people change at different rates over time
var(Residual)	107.570			High within-person (measurement or natural) variability
Interaction:	chi2(2) = 4.29, p = 0.1172			
LR test vs. linear model:	chi2(2) = 81.54, p < 0.0000			

Supplemental Table 6. Repeated measurements of Thyroglobulin (ng/mL).

LT4	Month	N	median	IQR25	IQR75
No	12	73	8.70	5.20	16.00
No	24	82	8.15	5.80	16.00
No	36	28	11.40	8.70	20.00
Early	12	16	5.60	3.75	23.00
Early	24	21	5.90	2.10	9.80
Early	36	10	5.65	4.00	32.00
Intermediate	12	10	10.10	5.20	16.00
Intermediate	24	8	3.80	2.40	7.35
Intermediate	36	4	0.94	0.19	4.45
Late	12	2	12.05	9.10	15.00
Late	24	1	8.90	8.90	8.90
Late	36	2	7.55	7.00	8.10

Supplemental Figure 1. Repeated measurements of FT4 (pmol/L)

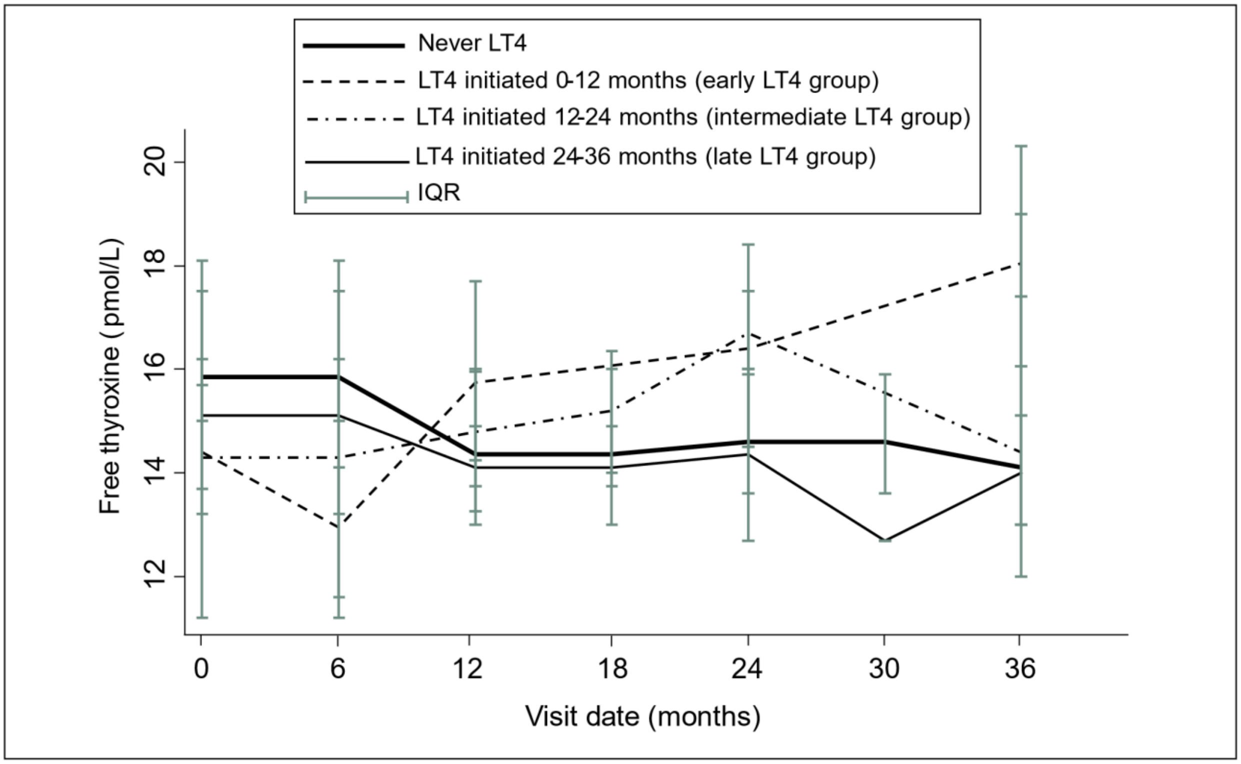

Supplement: Supplementary file 1 [file supplementary_materials.pdf]
